# Ulcerative colitis and irritable bowel patients exhibit distinct abnormalities of the gut microbiota

**DOI:** 10.1186/1471-230X-10-134

**Published:** 2010-11-12

**Authors:** Samah O Noor, Karyn Ridgway, Louise Scovell, E Katherine Kemsley, Elizabeth K Lund, Crawford Jamieson, Ian T Johnson, Arjan Narbad

**Affiliations:** 1 Institute of Food Research, Norwich Research Park, NR4 7UA, Norwich, UK; 2 Norfolk and Norwich University Hospital, Norwich, UK

## Abstract

**Background:**

Previous studies suggest a link between gut microbiota and the development of ulcerative colitis (UC) and irritable bowel syndrome (IBS). Our aim was to investigate any quantitative differences in faecal bacterial compositions in UC and IBS patients compared to healthy controls, and to identify individual bacterial species that contribute to these differences.

**Methods:**

Faecal microbiota of 13 UC patients, 11 IBS patients and 22 healthy volunteers were analysed by PCR-Denaturing Gradient Gel Electrophoresis (DGGE) using universal and Bacteroides specific primers. The data obtained were normalized using in-house developed statistical method and interrogated by multivariate approaches. The differentiated bands were excised and identified by sequencing the V3 region of the 16S rRNA genes.

**Results:**

Band profiles revealed that number of predominant faecal bacteria were significantly different between UC, IBS and control group (p < 10^-4^). By assessing the mean band numbers in UC (37 ± 5) and IBS (39 ± 6), compared to the controls (45 ± 3), a significant decrease in bacterial species is suggested (p = 0.01). There were no significant differences between IBS and UC. Biodiversity of the bacterial species was significantly lower in UC (μ = 2.94, σ = 0.29) and IBS patients (μ = 2.90, σ = 0.38) than controls (μ = 3.25, σ = 0.16; p = 0.01). Moreover, similarity indices revealed greater biological variability of predominant bacteria in UC and IBS compared to the controls (median Dice coefficients 76.1% (IQR 70.9 - 83.1), 73.8% (IQR 67.0 - 77.5) and 82.9% (IQR 79.1 - 86.7) respectively). DNA sequencing of discriminating bands suggest that the presence of *Bacteroides vulgatus, B. ovatus, B. uniformis*, and *Parabacteroides sp*. in healthy volunteers distinguishes them from IBS and UC patients. DGGE profiles of Bacteroides species revealed a decrease of Bacteroides community in UC relative to IBS and controls.

**Conclusion:**

Molecular profiling of faecal bacteria revealed abnormalities of intestinal microbiota in UC and IBS patients, while different patterns of Bacteroides species loss in particular, were associated with UC and IBS.

## Background

Microbial populations in the gut are known to play an important role in the health of the human gastrointestinal (GI) tract and provide an efficient barrier against invading gastrointestinal pathogens [1]. Little is known, however, about the contributions of particular intestinal species to health and disease. Irritable bowel syndrome (IBS) and ulcerative colitis (UC) are two very different disorders of the GI tract which share some common symptoms such as pain and alteration of bowel habits. The causes of UC and IBS are still not clear, but it is generally accepted that both are multifactorial in origin, and that host genetics [2,3], environmental factors [4,5] and unregulated immune responses [6,7] are involved. Moreover most studies suggest that the gut microbiota is an important factor in the pathogenesis of both UC and IBS.

A number of recent studies confirm the involvement of gut bacteria in the aetiology of ulcerative colitis. For example, HLA-B27 transgenic rats used as an animal model for studying human inflammatory disorders do not develop inflammation in the small or large bowels when kept in a germ-free environment [8]. However when germ-free HLA-B27 rats were reconstituted with normal luminal bacteria they developed gut inflammation [9]. In humans, infectious diarrhoea caused by *Salmonella, Shigella *or campylobacter was followed by IBS in seven to thirty per cent of patients [10,11]. The instability of intestinal microbiota reported in IBS [12], combined with the abnormal colonic fermentation [13], and mitigation of IBS symptoms by controlling small intestinal bacterial overgrowth by antibiotic therapy [14,15], suggest that intestinal microbiota may also be involved in IBS. Consequently, there is growing interest in treating both IBD and IBS by influencing the composition and activities of the resident microbiota. For example, a short term intervention with a symbiotic preparation revealed improvement in inflammation indices in patients with active UC [16], and administration of a probiotic mixture resulted in improved IBS symptoms in a six-month controlled intervention study [17]. A number of publications have reported a decrease of bacteria belonging to the groups *Clostridium *XIVa and *Clostridium *IV in patients with Crohn's disease (CD), [18-20]. In another study a reduction of *Faecalibacterium prausnitzii *an important member of mucosa-associated Firmicutes was found to be associated with the recurrence of ileal CD [21]. The authors also reported that a secreted product of *F. prausnitzii*, had an immunomodulatory activity *in vitro*, and that oral administration of this bacterial species or its supernatant can reduce the severity of experimental colitis in mice.

However, a better understanding of which bacteria or bacterial populations are relevant to UC and IBS is an important prerequisite if microbial interventions are to be used in preventing or managing these conditions. Few studies have investigated faecal microbiota of UC and IBS patients using quantitative techniques under comparable conditions. Such studies are technically difficult because more than 1000 bacteria species have been reported in the human colon to date, and large per cent of those bacteria remain uncultured [22]. Molecular techniques overcome these difficulties by allowing complex bacterial communities to be characterised and quantified. In the present study we have used the culture-independent technique, PCR-denaturing gradient gel electrophoresis (PCR-DGGE) of the V3 region of the 16S rRNA gene, to investigate the gut microbiota of UC and IBS patients as well as healthy controls. DGGE is a powerful technique for studying the complex microbial communities. It has been extensively used in studies of gut bacteria, including those designed to better understand changes in gut microbiota due to environmental disturbance [23]. DGGE has advantages over other molecular approaches in that it is a high throughput, relatively inexpensive method that allows detailed characterisation of predominant bacteria by cutting the individual bands, re-amplifying and sequencing, without the need for a cloning procedure. The aim of the present study was therefore to use this approach combined with unique in-house developed method for normalisation of DGGE data to investigate how bacterial populations differed between healthy people and those with UC and IBS and identify the bacterial species which are responsible for these differences. Our results revealed abnormalities of intestinal microbiota in UC and IBS patients, but also more interestingly we observed a loss of specific Bacteroides species.

## Methods

### Subjects and sample collection

UC and IBS patients were recruited via the Gastroenterology Clinics at the Norfolk and Norwich University Hospital (Norwich, UK). Healthy controls were recruited through the Institute of Food Research (IFR) Human Nutrition Unit (HNU). A total of 13 UC patients (6 males, 7 females), 11 IBS patients (4 males, 7 females) and 22 controls with no gastrointestinal symptoms (5 males, 17 females), were recruited over a period of 13 months between June 07 - July 08. The median (range) ages were 45 (23-64) years for UC group, 45 (25-64) years for the IBS group, and 45 (21-61) years for controls. All patients were anonymous and in remission when samples were collected. All UC patients had a disease activity score in the moderate to severe range when recruited. Participants in the study provided written consent prior to the completion of a health questionnaire. Infectious diseases and structural abnormalities of the gastrointestinal tract were excluded in all subjects. Each patient had a diagnosis of IBS (ROME II criteria) or UC (Mayo Clinic criteria >2) [24] made by their clinician. Volunteers enrolled in the study had not taken probiotics or prebiotics in any form in the previous two weeks, and had not received any antibiotics within 4 weeks before providing their samples. Our study was approved by the Institute of Food Research Human Research Governance Committee (HRGC), East Norfolk & Waveney Research Governance Committee and the Suffolk Research Ethics Committee (Suffolk REC), project ref (06-Q0102-91).

The methods of sample collection and storage were explained to the participants, and written notes were given with the stool collection pots, biohazard bags, and ice cube bags. Volunteers were asked to deliver the samples to the laboratory within 2 h of defecation.

### Extraction of total DNA from stool samples

Bacterial DNA was extracted from 0.2 g aliquots of frozen faecal sample using QIAamp Stool Mini Kit (Qiagen, West Sussex, UK) according to the manufacturer's instructions. The purity and concentration of the extracted DNA were measured using a spectrophotometer at 260 and 280 nm (ND, Nanodrop Technology, Wilmington, USA). The integrity of genomic DNA was also visualized following electrophoresis using a 1% agarose gel.

### PCR amplification of 16S rRNA genes

DNA isolated from faecal material was used as a template in PCR amplification. PCR was performed using universal primers, 341GC-f and 534-r which target the variable V3 region of the 16S rRNA gene. PCR was performed as previously described by Muyzer [25]. The specific primers Bac32GC-f (5'-CGCCCGGGGCGCGCCCCGGGCGGG GCGGGGGCACGGGGGGAACGCTAGCTACAGGCTT-3') and 303-r (5'-CCAATGTGGGGGACC-3') were also used to amplify the V3 region of the bacteria belonging to the Bacteroides group. The PCR products were checked on 1.5% agarose gel to confirm that a single band of the expected size was obtained. PCR products were then purified using SureClean (Bioline, London, UK) and DNA concentration was again measured by spectrophotometer (ND, Nanodrop Technology, Wilmington, USA).

### DGGE

DGGE was performed using the D-Code system (Bio-Rad Laboratories, UK) according to the manufacturer's instructions. DGGE with 8% polyacrylamide gel containing 40-58% linear denaturant gradient (100% denaturant corresponds to 7 M urea and 4% deionised formamide) was used. Sub-samples (150 ng) of each purified PCR product were loaded onto the gel in duplicate. Each sample was run on at least two different gels. Replication was performed so that any faulty or otherwise outlying lanes could be excluded from the subsequent data analysis, without compromising the total number of independent samples from which measurements were available. In addition, a synthetic standard (SS) was loaded in at least 4 positions onto each gel, in all cases including the outermost lanes. The SS is a mixture of different samples designed to produce a profile with a high number of bands spread across the full length of gel. The band positions were used as reference points in the lane alignment procedure outlined below. DGGE was performed at 60˚C and 50V for 18 hrs in 0.5X TAE buffer. Gels were stained with SYBR green I (1:20000 in 0.5X TAE) and then visualised using a molecular imager system (Pharos-FX-plus, BioRad, UK) with external laser modules (BioRad, UK).

### DGGE profile analysis

DGGE images were analysed by TotalLab120 software for gel images (Nonlinear Dynamics, Newcastle, UK). This package was used to correct the gel images for gross distortion, and to export a set of column vectors representing the greyscale intensity of each lane.

All subsequent data analysis was carried out using Matlab^® ^version 6.1 installed with the Statistics and Image Processing toolboxes (The Math Works Inc, Natick, Massachusetts, USA). Routines for performing within- and between-gel alignment were developed in-house, as follows. The positions of the SS band maxima were identified (by peak-picking) on all gels. To remove any remaining band curvature resulting from experimental variation, polynomial fitting of the SS band positions within each gel was used to generate a correction factor for all lanes on that gel. To achieve between-gel alignment, piecewise linear interpolation was used to stretch or shrink each block of data bounded by SS bands, using the global mean of the SS band positions as the index limits in each case. This procedure is similar to a previously reported method for aligning PCR-DGGE gel profiles [26]. However we note that the inclusion of multiple replicate lanes per gel in the present study substantially eases the task of registration.

After alignment, the data from each sample comprises a single vector containing 1001 elements. This length was chosen so that the indices map easily onto a 0 to 1 scale with increments of 0.001, corresponding to the conventional "RF" scale used in DGGE. Note that the original images all contained somewhat more than 1000 pixels along each lane. After visual examination of the full data matrix (viewed as a greyscale image), it was decided to truncate each lane to 731 elements corresponding to the RF range 0.09 - 0.82. The discarded regions (from the top and bottom of the gels) were found to be highly variable. A number of visually poor quality lanes were also discarded. Finally, data from each lane were scaled so that all elements were in the range 0 to 1, to mitigate the effect of between-gel intensity variance.

The resulting [96 × 731] data matrix was processed by the following multivariate methods: Partial Least Squares Discriminant Analysis (PLS-DA) was carried out to compress the raw data to a smaller number of variables, and to look for evidence of grouping according to disease state ("Y" variable is the class membership). Canonical Variate Analysis (CVA) was used to obtain an optimal 2-dimensional representation of the group structure [27]. All multivariate modelling was implemented using leave-sample-out cross-validation throughout.

Estimates of the numbers of discrete bands in each lane were made by peak-picking subject to a user-defined threshold. Weighted biodiversity of dominant microbiota was also calculated according to the Shannon index of diversity which simultaneously takes into account both the number of bands and the band intensities [28,29]. Similarity indices were calculated using the Dice coefficient, with an appropriate tolerance (+/- 0.008) included on the Rf scale. Where duplicate (or even triplicate) measurements existed for a given sample, these data were averaged before further statistical analysis. Where parametric statistical tests are used, data were assessed in advance for normality and equality of variance.

Selected bands corresponding to the highest peak maxima generated by PLS analysis were excised from the gels, re-amplified, and sequenced using the corresponding V3 region primers. The sequencing reactions were performed with BigDye v.3.1 (Applied Biosystems, UK), and checked using DNAStar programme (SeqMan). Resulting DNA sequences were compared with other 16s rRNA gene sequences in the GenBank database at the National Centre for Biotechnology Information (NCBI).

## Results

**Figure 1:**
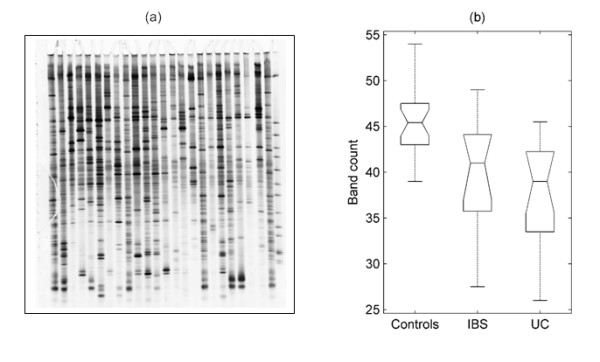
**DGGE profiles and band numbers**. (a) DGGE profiles of faecal samples obtained from IBS, UC and control subjects showing the number of bands corresponding to amplicons of the V3 region of 16S rRNA of faecal bacteria. (b) Box plots presenting the median and range of band counts of all faecal samples taken from UC, IBS, and controls. (Boxes have lines at the lower quartile, median, and upper quartile values; whiskers show extent of the rest of the data. Notches display the variability of the median between samples; notches that do not overlap have different medians at the 5% significance level).

**Figure 2:**
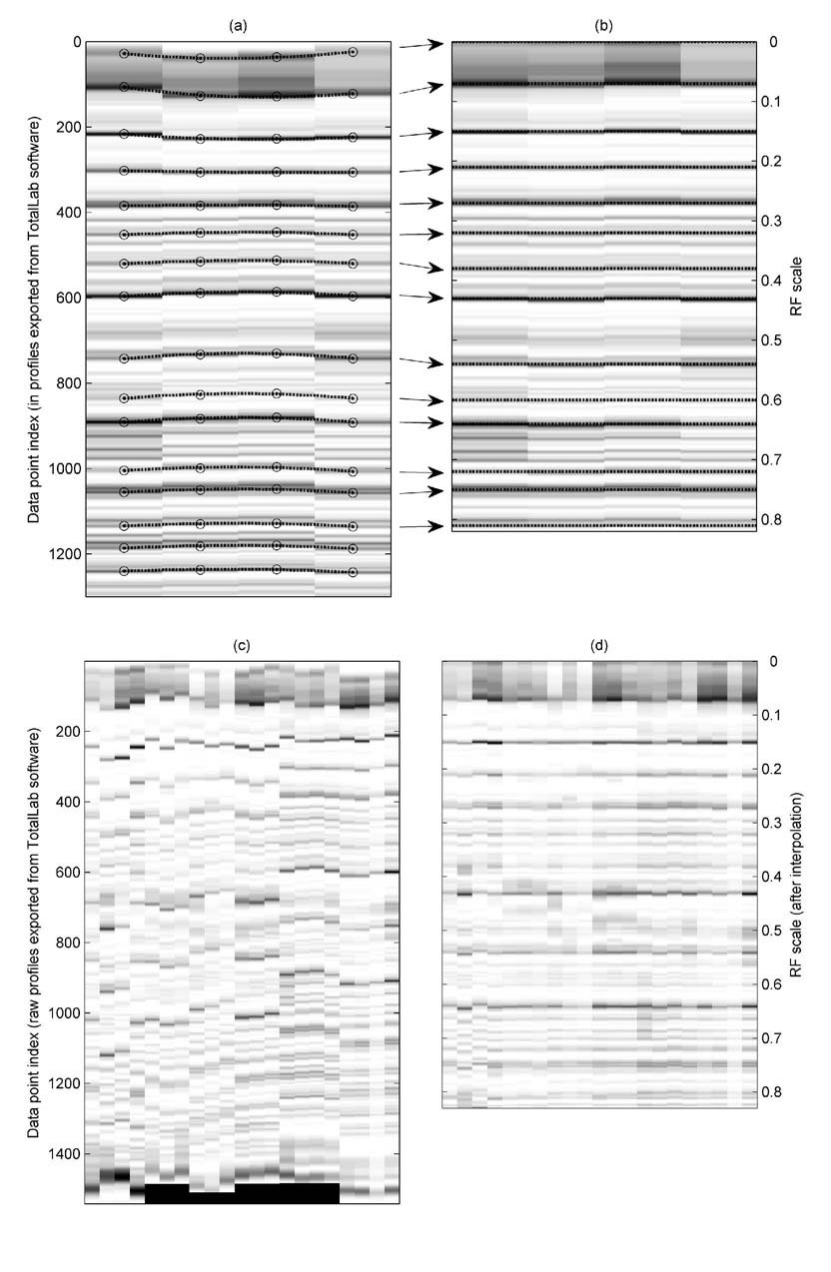
**Synthetic Standards (SS) alignment**. SS lanes from a single typical gel, (a) before alignment and (b) after alignment, showing relative stretching and shrinking of piecewise regions in the bespoke alignment routine. Images of all SS lanes from all gels: (c) before, and (d) after the complete alignment procedure.

All samples were analysed using DGGE to determine the biodiversity of gut microbiota of UC and IBS patients, as well as healthy controls. The PCR product contained a mixture of gene amplicons of the same size from different bacterial species that could be separated according to the sequence differences of the variable V3 region in the 16S rRNA gene. On DGGE, each sample produced band patterns reflecting the predominant bacterial communities in the samples (Figure 1). Once DGGE patterns were generated, all subsequent data were aligned and normalised in Matlab. The effect of the pre-treatment (alignment and normalisation) is illustrated in Figure 2 using the synthetic standards demonstrating that the method employed allows accurate alignment of bands both within a single gel but also of much pronounced variations in band positioning observed when comparing profiles within different gels. A number of different analytical techniques were then applied to interrogate the data. First, the microbial diversity was estimated by the numbers of bands present in the DGGE patterns (Figure 1b). Controls showed higher number of bands (45 ± 3) compared to UC (37 ± 5) and IBS patients (39 ± 6) indicating reduced diversity of the predominant bacteria in both patient groups. One-way ANOVA confirmed (p < 10^-4^) that the groups do not have the same mean band count. The lower band counts found in the latter groups indicate the possible absence or reduction in the abundance of specific bacterial classes amongst their predominant bacteria compared with healthy controls. The means were compared pairwise using a multiple comparison test. The control group was found to differ significantly from both the IBS and UC groups (at the p = 0.01 level, with Bonferroni adjustment). No significant difference was found between the IBS and UC groups.

Similar findings are obtained from examination of the Shannon indices, which make use of band intensity as well as count data. One-way ANOVA again confirmed (p < 10^-3^) a significant difference between the means, with multiple comparisons indicating that the mean Shannon index is significantly (at the p = 0.01 level, with Bonferroni adjustment) higher in controls (μ = 3.25, σ = 0.16) compared with the UC (μ = 2.94, σ = 0.29) and IBS (μ = 2.90, σ = 0.38) groups.

The pairwise similarity data were assessed using non-parametric, descriptive methods only, since independence and distributional considerations preclude the use of standard techniques. The controls had a median similarity of 82.9% (IQR 79.1 - 86.7), followed by UC patients who shared 76.1% (IQR 70.9 - 83.1) of their predominant faecal bacteria. IBS individuals had median similarity of only 73.8% (IQR 67.0 - 77.5).

**Figure 3:**
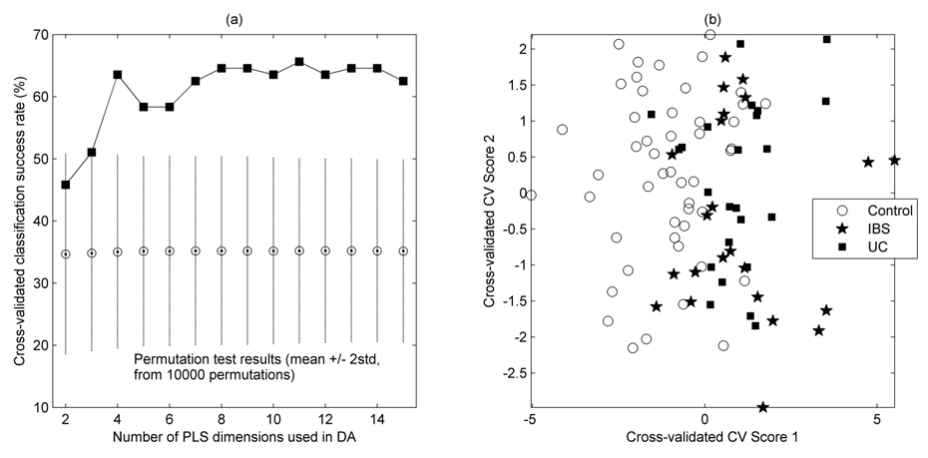
**Statistical analysis of DGGE profiles**. (a) Classification success rate (cross-validated) versus number of PLS dimensions used. A local maximum of ~64% was obtained from a 4-factor model. Also shown are compact boxplots summarizing the results of PLS-DA permutation tests (using y-scrambling, 10,000 re-samples). (b) CVA scores plot derived from PLS scores (4 dimensions) of the DGGE data of faecal bacteria showing the relative positions of the UC, IBS, and control groups. (NB: fully-leave-sample-out cross-validated.)

PLS-DA was used to obtain a model for classifying observations according to the disease state. Note that the cross-validation implemented was leave-sample-out. That is, replicate lane data were always grouped together into the cross-validation segments, to prevent over-fitting the model. The classification success rate summarized across cross-validation segments is shown in Figure 3a as a function of the number of PLS scores used. The first local maximum in the success rate (of ~64% correct) is obtained from 4 PLS dimensions. Further inspection showed that the majority of the incorrect assignments were of the two disease states; in other words, the model is most able to distinguish between controls and disease, and less able to separate the two disease states. A permutation test ("y-scrambling") was carried out to assess whether this success rate was likely to be a chance outcome (the range of classification results obtained under the null hypothesis are indicated by the bars on Figure 3a, from which we estimate a p-value of < 10^-6^. We can conclude that there is a strong evidence of grouping present in our data. To obtain a visual illustration of the classification model, PLS-DA was combined with CVA, since this provides, by definition, the best 2-dimensional representation of a 3-group structure. In view of the success rate behaviour, 4 PLS dimensions were considered to yield a parsimonious model, and these scores were passed to the CVA routine (again implemented in full leave-sample-out cross-validated form). We see (Figure 3b) that both the UC and IBS groups are readily distinguished from the controls, supporting the premise that there are systematic differences in the microbial diversity particularly between healthy and diseased (UC and IBS) volunteers. The profiles of the IBS patients appear to be somewhat more dispersed, possibly indicating even greater variation amongst their microbial populations, and consistent with the band-count findings reported above.

**Figure 4:**
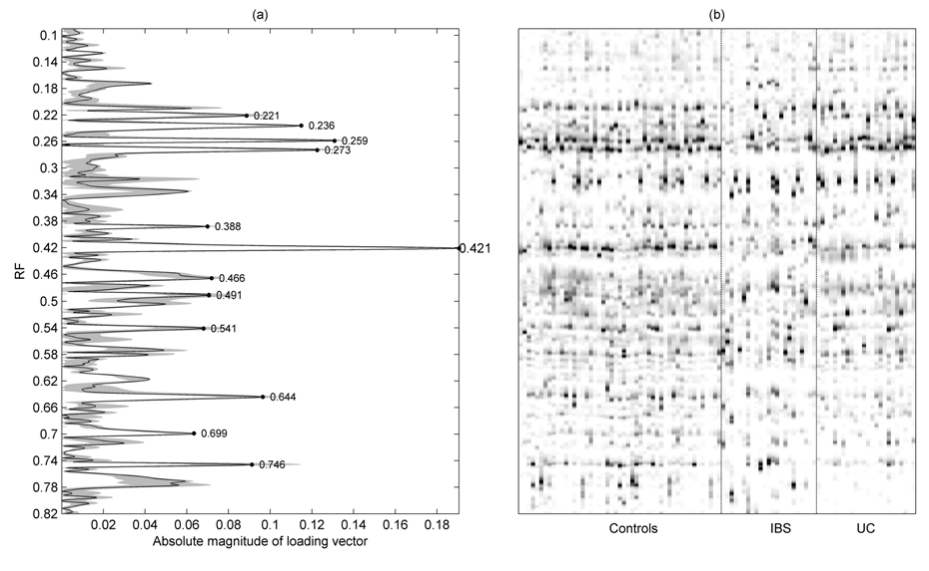
**Bands analysis of DGGE profiles**. (a) Absolute values of first PLS loading, peak-picked to show bands that are most responsible for distinguishing between the groups. Greyed area indicates range of this loading across the training segments. (b) Greyscale image view of complete aligned dataset, on the same Rf axis.

**Figure 5:**
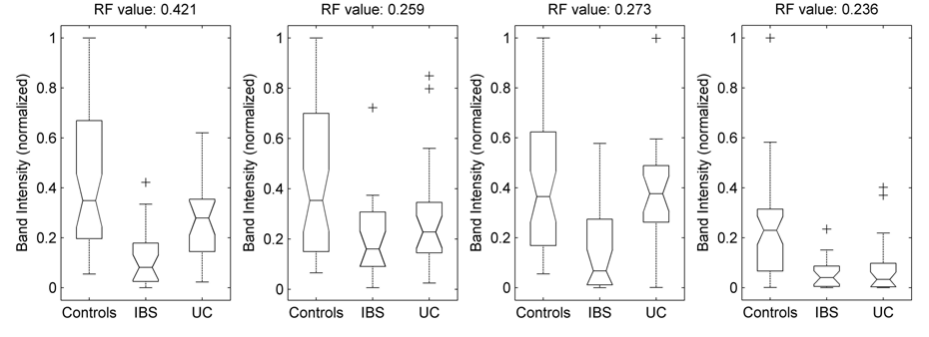
**Top-ranked discriminating bands**. Boxplots summarizing the normalized intensities of the four top-ranked discriminating bands, measured separately for each of the three groups.

An output of the PLS analysis is a set of "loading vectors". The magnitudes of the vector elements can be plotted as series of peaks, the heights of which indicate the relative contribution of each band to the successive separations between groups. Inspection of the PLS scores (for conciseness, not shown) indicated that the first PLS dimension is primarily responsible for separating the diseased from healthy individuals. Figure 4a shows the mean of this vector obtained from all training segments, alongside an image view of the full data matrix (Figure 4b). Some of the top-ranked features are peak-picked. The stability of the loading with respect to the cross-validation is indicated by the solid greyscale area, which shows the range obtained. The four top-ranked bands were consistent across all cross-validation passes, hence these were selected for further experimental investigation, that is, they were excised from the gel and sequenced. Analysis of the V3 region of the 16S gene sequences revealed that the excised bands were all members of the genus *Bacteroides *and *Parabacteroides *(table 1). It is interesting that these bands were more frequently observed in the healthy controls than in UC and IBS (Figure 5), apart from one band (*Bacteroides vulgatus*) which was found in the UC patients but not in IBS patients.

**Table 1:** The identified top-ranked bands selected and excised from DGGE gel.

Rf Value	Taxonomic affiliation	% similarity
0.421	*Bacteroides uniformis*	97%
0.259	*Bacteroides ovatus*	93%
0.273	*Bacteroides vulgatus*	99%
0.236	*Parabacteroides distasonis*	82%

The V3 regions of the 16S rRNA gene sequences were compared with those in GenBank, National Centre for Biotechnology Information (NCBI)

**Figure 6:**
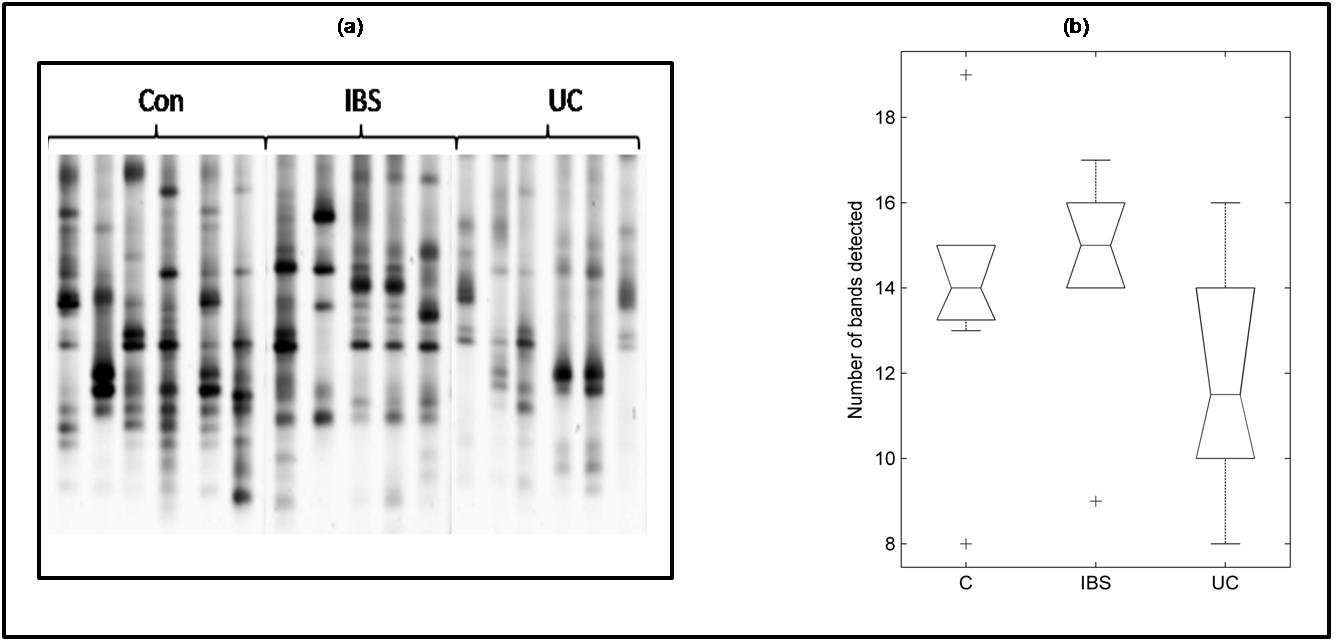
**Bacteroides specific DGGE profiles**. (a) Example of DGGE profiles obtained with Bacteroides specific primes in faecal samples obtained from IBS, UC and control subjects. (b) Boxplots showing band numbers in DGGE profiles of faecal samples taken from UC, IBS, and control.

In order to further investigate the observed differences in bacteroides composition, we performed DGGE analysis using only the bacteriodes specific primers. Results shown in Figure 6 indicate that there were differences in the diversity of bacteroides species between the healthy and the patient groups. The mean numbers of bands detected in each sample were as follows: controls, μ = 14.0 (σ = 3.3); IBS, μ = 14.3 (σ = 2.9); and UC, μ = 11.9 (σ = 2.9). Note, however, that these differences were not statistically significant, possibly due to the relatively small numbers of samples involved here. The median similarities between bacteroides profiles were as follows: controls, 58.8% (IQR 51.0 - 70.1); IBS, 55.2% (IQR 48.9 - 65.6) and UC, 36.4% (IQ 24.5 - 49.0).

## Discussion

Previous studies have suggested that abnormalities of the colonic microbiota occur in both UC and IBS [30,31]. However the putative ability of certain bacterial species to enhance gut health, and the practicality of intervention strategies such as the use of prebiotics to achieve this, remains uncertain and poorly understood. Previous investigations of faecal or of biopsy-associated bacteria have failed to identify particular pathogens or bacterial groups linked to the altered microbial composition in UC patients relative to control subjects [30,31]. However the application of molecular profiling techniques allows characterisation of the unculturable bacteria in the human colon, and in the present study has enabled us to address this issue in greater detail. Using this approach we have confirmed the existence of significant abnormalities in the predominant faecal bacteria of UC and IBS patients in relation to controls, using qualitative and semi-quantitative DGGE analysis, isolation and identification of the missing phylotypes.

DGGE is a semi-quantitative technique which provides a snapshot of the predominant bacterial species in a complex ecosystem such as human colon. Many of the studies to date have monitored changes in DGGE patterns by comparing the presence and absence of bands, or changes in the intensity of a single band on the same gel [32]. Other studies have used pair-wise similarity coefficients, based on common bands shared between different samples [33]. The total band numbers in a given sample, and their intensities, were combined to assess differences between DGGE profiles [34,35]. However, human colon has a very complex bacterial community, often resulting in large number of bands, and furthermore the co-migration of the same bands on different gels can often make accurate analysis of DGGE data difficult [36]. In our study we have applied a careful alignment method using the synthetic standards to accurately align bands between gels to mitigate experimental variation. The duplicate runs on different gels have given us additional confidence in the alignment procedure. To obtain a semi-quantitative DGGE analysis, the total number of bands and their intensities were included in our analysis after normalization.

Clinical and experimental observations have suggested the involvement of intestinal bacteria in the pathophysiology of UC; however the mechanisms by which bacteria or disease-specific pathogen are involved in UC have yet to be determined [37]. Our data showed significant differences in the predominant faecal bacteria in UC patients relative to controls, suggesting a loss or reduction of bacterial classes, leading to the possibility of phylogenetic changes in these patients. This observation is consistent with other recent studies which reported a 40% decrease of dominant bacteria in UC patients [38]. Such a decrease might be a response to environmental influences such as changes in diet or drugs or to endogenous signals derived from inflammatory changes or the immune status.

In a previous study, the predominant faecal bacteria of UC patients under remission were shown to be less stable than those of healthy controls, and the results revealed a host-specific pattern in relation to predominant bacteria in UC individual [39]. Studies based on DGGE have shown small fluctuations of around 20% in predominant bacteria in healthy controls [40]. Our results indicated that healthy controls share around 78% of predominant faecal bacteria, suggesting that this stability is a feature of a healthy gut. In contrast, UC individuals shared only around 70% of their predominant bacteria, which indicates that a particular shift of bacterial community might be a feature of UC patients. IBS patients shared around 68% of gut predominant species, and thus also displayed an even more diverse bacterial community amongst individuals, compared to controls. This observation indicates that despite the absence of inflammatory activity, this patient group is also disposed to changes affecting the composition of their faecal bacterial community. The reduced microbial diversity seen here with UC and IBS patients is, in agreement with the findings of previous studies. Martinez et al [39] demonstrated reduced bacterial diversity of faecal bacteria from patients with active UC using a similar approach to that reported here. Ott et al [28] also reported a reduction in bacterial diversity in tissue biopsies from UC and Crohn's disease patients using polymorphism analysis of amplicons of 16S rRNA. Our study has expanded our understanding of this phenomenon by undertaking qualitative analysis of the missing genera.

The GI microbiota is also thought to play an important role in the development and persistence of IBS; however, the numbers of detailed studies using culture-independent profiling methods are limited. Using classical methods, Balsari et al. [41] reported qualitatively similar faecal bacteria in IBS patients and healthy controls, with some quantitative differences in the predominant bacteria. In contrast, our results show that the microbiota of IBS patients is quantitatively different compared to those of UC or the healthy controls, and that the inter-individual variation in predominant microbiota composition was greatest amongst the IBS patients. This observation might reflect the fact that IBS is a poorly defined condition not associated with any single, well defined biochemical, structural, or serologic abnormality. Thus although we have based our diagnoses on Rome II (a symptom-based criterion) to identify IBS patients, we are still likely to be sampling IBS patients from different sub-groups. Typical symptoms of IBS vary from constipation to diarrhoea, and both may be present at different times in the same patient. Therefore, it is sensible to assume that differences in intestinal bacteria within the global IBS group could be associated with the IBS subgroups. Such an interpretation is consistent with the observations of Malinen et al [30] who used RT-PCR to analyse faecal bacteria of IBS individuals and reported changes in different bacterial spp. related to IBS subgroups exhibiting different symptoms. We did not attempt to differentiate the gut flora in IBS subgroups since the total numbers of volunteers was relatively small.

It has been shown by others that 3% of patients with enteric infections can develop UC [42]. However, this figure is much lower than the 10% who develop IBS after infection [43]. Hence, it is interesting to note that in the present study the predominant faecal bacterial population from some IBS patients more closely mirrors those of UC patients, and it would therefore be of interest to assess whether such patients might represent a post-infection IBS sub-group, and if they are at a higher risk of developing UC.

The members of the genus Bacteroides are one of the most frequently represented bacterial species in the human colon [44]. A few species, such as *B. fragilis*, are considered to be human pathogens, but most are thought to be normal commensal bacteria. In animal models of IBD, supplementation of the normal intestinal bacteria with a dose of *Bacteroides vulgatus *has been reported to worsen the condition [45,46]. Furthermore, it has been suggested that *Bacteroides *is involved in the reoccurrence of UC after surgery [47], although this study was small scale and so further evidence is needed. In the present study, despite the fact that there was no specific band found to be associated with UC or IBS patients, we were able to establish that *Bacteroides *spp. were most responsible for group discrimination. Surprisingly, and contrary to our expectations, our data revealed that *Bacteroides vulgatus*, probably *Bacteroides ovatus, Bacteroides uniformis*, and *Parabacteroides sp*. were more commonly present at higher levels in healthy controls than in UC or IBS patients.

In this context it is interesting that an earlier study demonstrated that *B. vulgatus *can protect against *E. coli*-associated colitis [48]. More recent observations by Sydora et al. suggested that *B. vulgatus *plays a role against the development of colitis [49]. Conte et al (2006) found that amongst mucosal associated bacteria, *B. vulgatus *occurrence was particularly low in patients with UC, Crohn's disease or with indeterminate colitis [50]. A recent report by Takaishi et al (2008) also indicates that that the level of *B. ovatus *was reduced in UC patients with active colitis [51]. *B. ovatus *has also been reported to protect germ-free and conventional mice, exposed chronically to dextran-sodium sulphate (DSS), from bleeding, development of intestinal inflammation and death [52].

## Conclusions

In summary, our findings demonstrate that there are significant alterations in the composition of gut microbiota in patients with UC and IBS compared to controls. As has been reported previously, we were able to confirm the reduction of microbial diversity in these patient groups. The reductions in some species of *Bacteroides *and possibly *Parabacteroides *in both conditions suggest a possible loss of protective role by this group of bacteria, rather than a specific organism playing a role in the pathogenesis of UC and IBS. However, an explanation for such a putative protective mechanism is still unidentified and requires further investigation.

## Abbreviations

UC: Ulcerative colitis; IBS: Irritable bowel syndrome PLS-DA: Partial Least Squares Discriminant Analysis; CVA: Canonical variate analysis

## Competing interests

The authors declare that they have no competing interests.

## Authors' contributions

SN: Carried out most of the practical experiments and assisted with patient recruitment and manuscript preparation. KR, LL: participated in study design and proposal writing. LS, JC: Ethics approval, patient recruitment, proposal and manuscript preparation. KK: Performed the statistical and data analysis. AN: Project conception, and participated in its design and coordination and manuscript preparation. IJ: Participated in drafting the manuscript. All authors read and approved the final manuscript.

## Pre-publication history

The pre-publication history for this paper can be accessed here:

http://www.biomedcentral.com/1471-230X/10/134/prepub
